# Phosphate release from myosin Va occurs after the initial powerstroke but before the secondary powerstroke associated with ADP-release

**DOI:** 10.1016/j.jbc.2026.113303

**Published:** 2026-06-29

**Authors:** Christopher P. Marang, Brent D. Scott, Christopher M. Yengo, Edward P. Debold

**Affiliations:** 1 Department of Chemical Engineering, Stanford University, Palo Alto, California, USA; 2 Department of Health Sciences, College of William and Mary, Williamsburg, Virginia, USA; 3 Department of Cell and Biological Systems, Penn State College of Medicine, Hershey, Pennsylvania, USA; 4 Department of Kinesiology, University of Massachusetts, Amherst, Massachusetts, USA

**Keywords:** myosin, powerstroke, phosphate, force generation, energy transdcution, ADP-release, Myosin V

## Abstract

Myosins transduce chemical energy into mechanical work, with key steps being the powerstroke and release of phosphate (P_i_) from the active site. This process is still poorly understood, in part, because the order of these events remains controversial. We used two independent approaches to determine if P_i_ was released before or after the initial powerstroke with a single-headed myosin Va in a laser trap assay, at 100 μM ATP. First, we determined the effect of P_i_, and resistive load, on the size of the primary powerstroke, secondary powerstroke (hitch) and the duration of the actomyosin binding event (*t*_on_). At low resistive load, 30 mM P_i_ did not alter the powerstroke, hitch or *t*_on_. However, at higher resistive loads P_i_ significantly (*p* < 0.05) reduced *t*_on_ and eliminated the hitch while the powerstroke was unaffected. The second approach utilized a myosin Va construct containing a mutation (S217A) that slows P_i_-release from the active site. The powerstroke and the hitch were unaffected at every resistive load at 0 mM P_i_, but the hitch and *t*_on_ were significantly reduced by 30 mM P_i_ at the highest resistive load. These observations suggest that the primary powerstroke occurs with P_i_ in active site, implying that P_i_-release follows the powerstroke. The observation that P_i_ eliminates the hitch at high resistive load suggests that P_i_-release, and therefore P_i_-rebinding, occurs between the primary powerstroke and hitch. Thus, these results suggest the order of the key events in the energy transduction pathway of a prototypical molecular motor.

Myosin Va is a prototypical molecular motor responsible for driving vesicular transport within the actin dense regions of cells (1, 2). It moves processively along an actin filament by transducing the chemical energy of ATP into mechanical work (3, 4), in the form of a powerstroke. The key events, in this highly conserved transduction process (5), are the generation of a powerstroke and the release of phosphate (P_i_) from the nucleotide-binding site (6, 7, 8). However, the relative timing of these events remains unclear and is the focus of a vigorous debate (7, 8, 9, 10, 11, 12, 13). Atomic structures of myosin suggest P_i_-release precedes the powerstroke (9, 14, 15), while evidence from functional assays suggest that the powerstroke precedes the release of P_i_ (8, 10, 11, 12). Gaining a clear understanding of the order of these events is critical for understanding how myosins, and related motor protein’s function (16, 17). This knowledge is crucial for the development of pharmaceutical therapies to modulate the force-generating capacity and kinetics of myosin in a host of deadly diseases (18, 19, 20).

The millisecond time resolution and nanometer spatial resolution of the single-molecule laser trap presents a powerful technique to resolve the order of these events in myosins (8, 21). This assay can be used to directly determine the effect of elevated levels of P_i_ on the size and rate of myosin’s powerstroke, as well as on the duration of the actomyosin bond (8, 12, 22, 23). If P_i_-release precedes the powerstroke, elevated P_i_ will prevent, or delay, the powerstroke because whenever a P_i_ is released from the active site, a new P_i_ molecule rapidly rebinds before a powerstroke can occur. Scott *et al.* (8) used this approach and found that 30 mM P_i_ did not affect the size or rate of the powerstroke of myosin Va. They observed a similar result using a mutation that slows P_i_-release from the active site (S217A). However, the lifetime of the actomyosin bond (*t*_on_) was not significantly reduced in the presence of 30 mM P_i_ in either construct; indeed only the longest duration binding events were affected (8). This suggests that P_i_ did not readily rebind to actomyosin under the conditions of this assay because the rebinding of P_i_ should accelerate detachment from actin (24). Further support for the low frequency of P_i_-rebinding comes from the observation that the secondary powerstroke or “hitch” associated with ADP-release (25, 26), was unaffected by the elevated levels of P_i_. This was likely because the trap stiffness used (0.04 pN nm^−1^) did not provide enough resistive load to promote P_i_-rebinding, which is strain dependent (6, 14, 27). Indeed, when Marang *et al.* (22) increased the stiffness of the optical traps to increase the resistive load on ensembles of myosin Va, the duration of binding events was significantly reduced in the presence of P_i_, and this effect was sharply dependent on the magnitude of the resistive load (22). However, the use of mini-ensembles of myosin Va molecules (∼7–10 myosin heads) made it difficult to determine how P_i_ affected the powerstroke and *t*_on_ of a single myosin molecule. Similarly, elevated levels of P_i_ more readily induced detachment of a dimeric construct of myosin Va from actin under increasing resistive load, but the complexities of the two-headed processive motion made it difficult to resolve the order of the powerstroke and P_i_ release of a single myosin head (28).

Woody *et al.* (12) attempted to address this issue by utilizing an ultra-fast version of the laser trap assay, capable of rapidly applying loads to myosin while bound to actin (29), using beta-cardiac myosin. By actively oscillating the bead–actin–bead assembly the time resolution improves from a few milliseconds down to tens of microseconds, enabling resolution of the weakly bound and pre-powerstroke states, as well as the rate of the powerstroke (29). In this assay 10 mM P_i_ had no effect on the size or rate of the powerstroke, leading them to conclude that the powerstroke precedes P_i_-release. However, the number of short and intermediate duration binding events, which did not generate a powerstroke, also increased. This could have been caused by a P_i_-induced increase in the frequency of detachment from the weakly-bound pre-powerstroke state (12). However, an alternative explanation (14) suggested that this finding could also be consistent with P_i_ rebinding to the active site in an ADP-bound state (AM.ADP) and prevented the powerstroke, which would be consistent with P_i_-release preceding the powerstroke. An additional complication was that at the [ATP] used (1 μm) P_i_ likely rebound to actomyosin in rigor rather than an AM.ADP state, which could explain why P_i_ prolonged rather than shortened *t*_on_. Thus, despite the improvement in the time resolution of the assay the results did not rule out a model in which P_i_-release precedes the powerstroke.

Therefore, we applied increasing resistive loads to myosin Va in a single molecule laser trap assay (Fig. 1, *A* and *B*) at 100 μm ATP to directly determine the effect of P_i_ on the powerstroke and *t*_on_ when P_i_ rebinds in an AM.ADP state to resolve the order of these events. Two independent approaches were used to keep P_i_ in the active site; first by increasing the [P_i_] to 30 mM and then by introducing a mutation into myosin Va known to slow P_i_-release (S217A).

**Figure 1:**
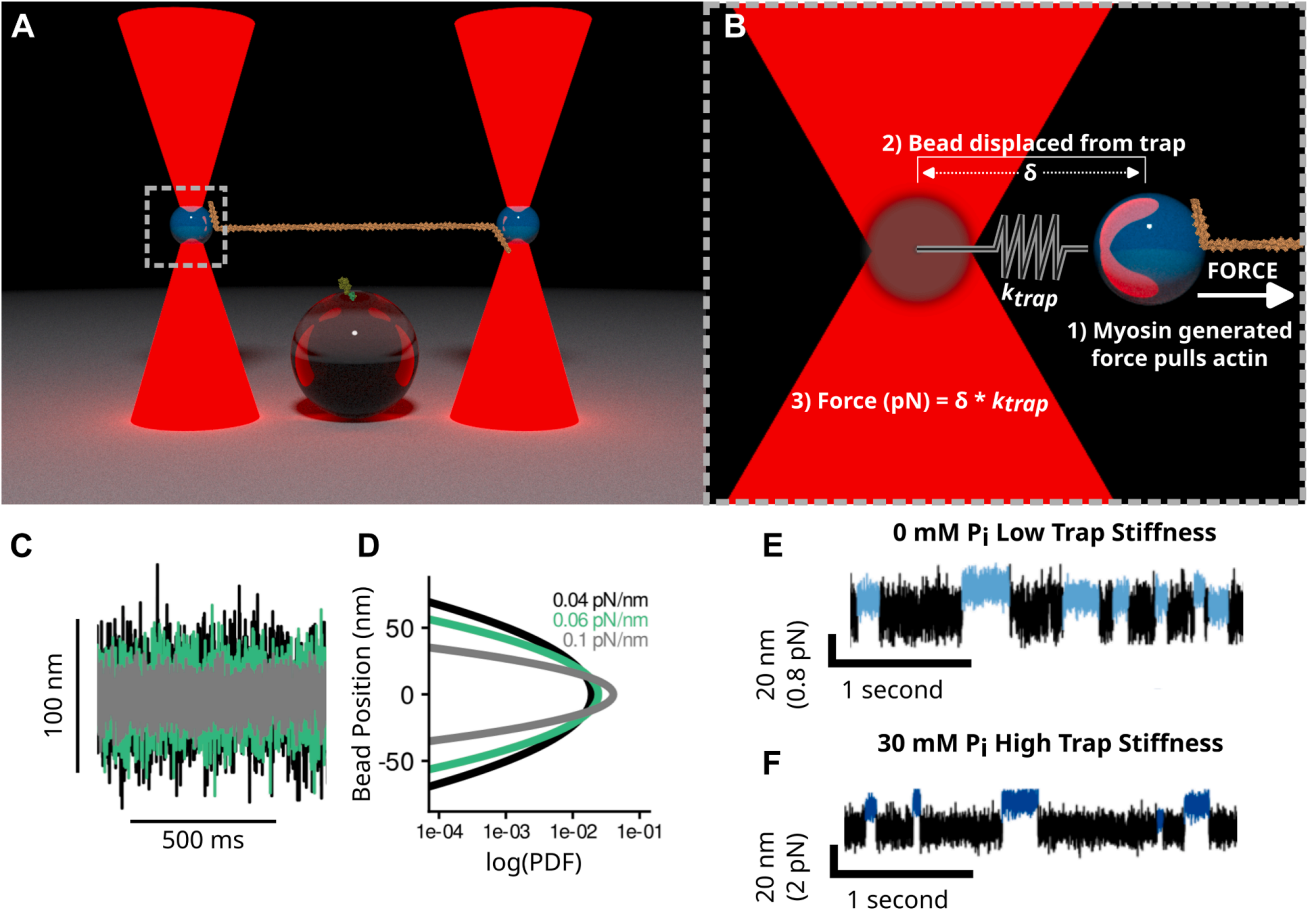
**Schematic** of the **laser trap assay.***A*, schematic of the laser trap assay showing the actin filament (*brown*) and myosin Va (*green*). *B*, the trapped bead behaves as if attached to a Hookean spring (i.e. F = k_trap_ ∗ x), where k_trap_ is the stiffness of the laser trap and δ is the distance that the bead is from the center of the trap. *C*, raw bead displacement records at increasing trap stiffnesses. *D*, bead position vs. power density function (PDF) for each trap stiffness, measure in the absence of actomyosin interactions. *E* and *F*, raw x-axis displacement records, detected events colored in *light* and *dark blue*.

## Results

By varying the intensity of the laser beam the stiffness of the laser traps was increased, which in turn increased the resistive force on myosin when it bound to, and displaced, the actin filament (Fig. 1, *B* and *C*). The increased resistive force provided the strain needed to facilitate P_i_-rebinding (6, 12, 30). Once bound to myosin’s active site, in an AM.ADP state, P_i_ rapidly weakens myosin’s affinity for actin and accelerates detachment (16, 31, 32) decreasing *t*_on_ (Fig. 1, *E* and *F*).

**Figure 2:**
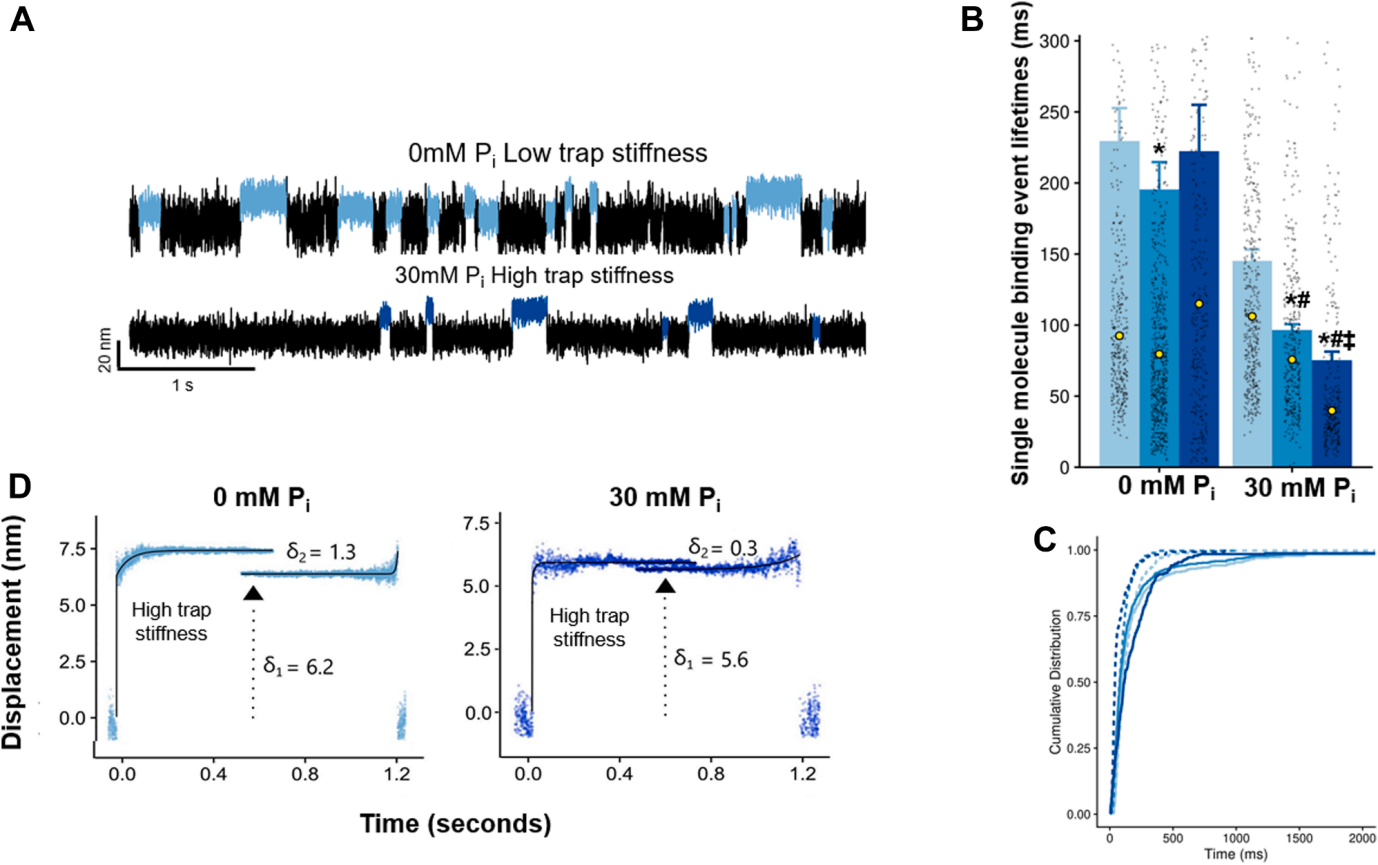
**Effect of P_i_ and trap stiffness on WT-myosin Va.***A*, representative raw bead displacement records. Colored sections indicate strongly-bound events, *black* sections indicate unbound periods. *B*, Bar graph of binding event lifetimes (*t*_on_) displaying mean ± SEM, and median (*yellow dot*), and individual binding events (*black dots*). *Left*-most bar (*light blue*) is the lowest trap stiffness (0.04 pN nm^−1^), middle bar (*medium blue*) is medium trap stiffness (0.06 pN nm^−1^), and the *darkest blue* corresponds to the highest trap stiffness (0.10 pN nm^−1^). The y-axis was truncated to show the shortest 80 to 90% of all events. ∗ significantly (*p* < 0.05) different from low stiffness at 0 mM P_i_, ^#^ significantly different from the lowest trap stiffness at 30 mM P_i_ (*light blue*). ^‡^ significantly different from medium stiffness (*medium blue*) with 30 mM P_i_. 99% of all events are displayed in the cumulative distributions, Figure 2*C*. Solid lines are in the absence of P_i_ and dashed lines in the presence of P_i_. Colors correspond to trap stiffness, same as 2B. Figure 2*D*) results of ensemble averaging, δ_1_ is the magnitude of the primary powerstroke and δ_2_ the size of the hitch. The rate of the primary powerstroke (k_0_) was 70,440^.^s^−1^ in the absence of P_i_ and 21,240^.^s^−1^ in the presence of 30 mM P_i_.

The low-trap stiffness data, which were previously published (8), served as a control for the medium and high-trap stiffness data (Fig. 2*A*). Increasing the trap stiffness had a minimal effect on *t*_on_ at 0 mM P_i_ but significantly reduced *t*_on_ at 30 mM P_i_ (Fig. 2*B*). Indeed, *t*_on_ was progressively reduced from low to medium to high-trap stiffness. Figure 2*C* shows 99% of the binding events as a cumulative distribution.

The start and end of each binding event were temporally aligned and then ensemble averaged to determine the effect on the powerstroke and the “hitch” or sub-step (Fig. 2*D*). In the absence of P_i_ the powerstroke was ∼6 nm, and the rate of transition from the weakly/unbound state into the powerstroke was >5000^.^s^−1^. Aligning of the ends of the binding events revealed a secondary powerstroke or hitch of ∼1 nm. The size of the first powerstroke and the hitch were unaffected by increasing the trap stiffness in the absence of P_i_, however, at high-trap stiffness, the hitch was reduced from 1.3 to 0.3 nm in the presence of 30 mM P_i_, (Fig. 2*D*). These values represent single-parameter estimates (33); therefore, this difference was not assessed for statistical significance.

**Figure 3:**
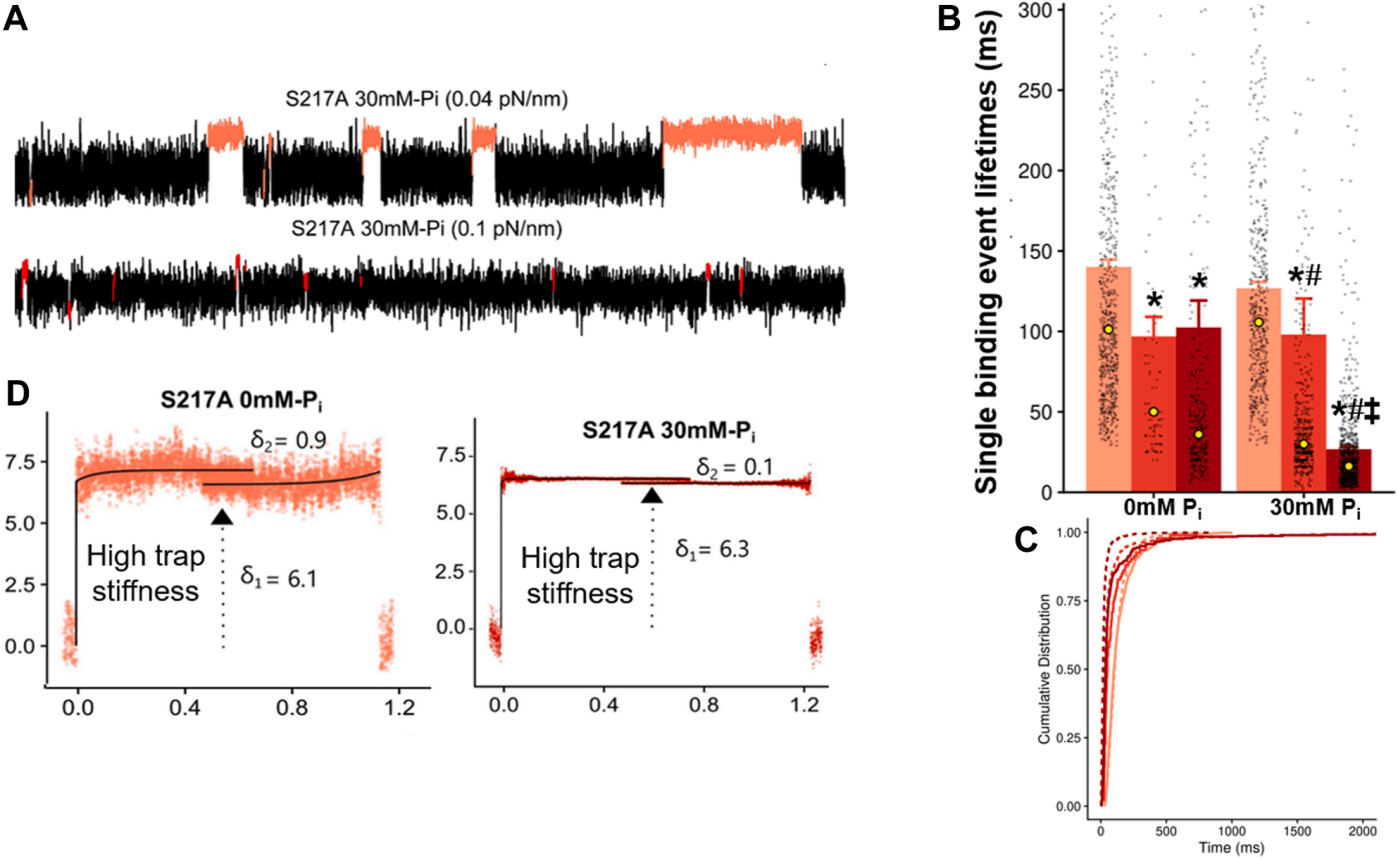
**Effect of P_i_ and trap stiffness on S217A-Myosin Va.***A*, representative raw displacement records. Colored sections indicate binding events and *black* sections unbound periods. *B*, graph of *t*_on_ displaying mean ± SEM, median (*yellow dot*) and individual binding events (*block dots*). *Left*-most bar (*lightorange*) is the lowest trap stiffness (0.04 pN nm^−1^), middle bar (*mediumorange*) medium trap stiffness (0.06 pN nm^−1^) and *darkestorange* the highest trap stiffness (0.10 pN nm^−1^). Y-axis was truncated to show the shortest 80 to 90% of binding events. ∗ indicates significantly different from low stiffness, 0 mM P_i_, ^#^ significantly different from high stiffness at 30 mM P_i_, and ^‡^ significantly different from medium stiffness at 30 mM P_i_. 99% of all events are displayed in the cumulative distributions shown in Figure 3*C*. *D*, results of ensemble averaging; δ_1_ size of the primary powerstroke and δ_2_ size of the secondary powerstroke or hitch. The rate of the primary powerstroke (k_0_) was 25,519^.^s^−1^ in the absence of P_i_ and 5211^.^s^−1^ in the presence of 30 mM P_i_.

A second set of experiments were performed using a myosin Va construct with a mutation (S217A) known to slow the rate of P_i_-release from the active site (9, 34, 35) (Fig. 3, *A*–C). Despite a slower P_i_-release rate the ensemble averaging analysis revealed that the size and rate of the powerstroke were identical to the WT construct (Fig. 3*D*). *t*_on_ was slightly shorter compared to WT in 0 mM P_i_ (Fig. 3*B*), likely due to the faster rate of ADP-release of this construct (34). We also exposed this construct to 30 mM P_i_ and found it was more dependent on the magnitude of the trap stiffness than WT (Fig. 3, *B* and *C*). For example, *t*_on_ was only significantly reduced by 30 mM P_i_ at the highest trap stiffness (Fig. 3*B*), but it was reduced by a significantly greater amount than the WT construct (Figs. 3*B* vs. 2*B*). As with the WT construct, the hitch was effectively eliminated at the highest stiffness in the presence of 30 mM P_i_ in S217A (Fig. 3*D*).

**Figure 4:**
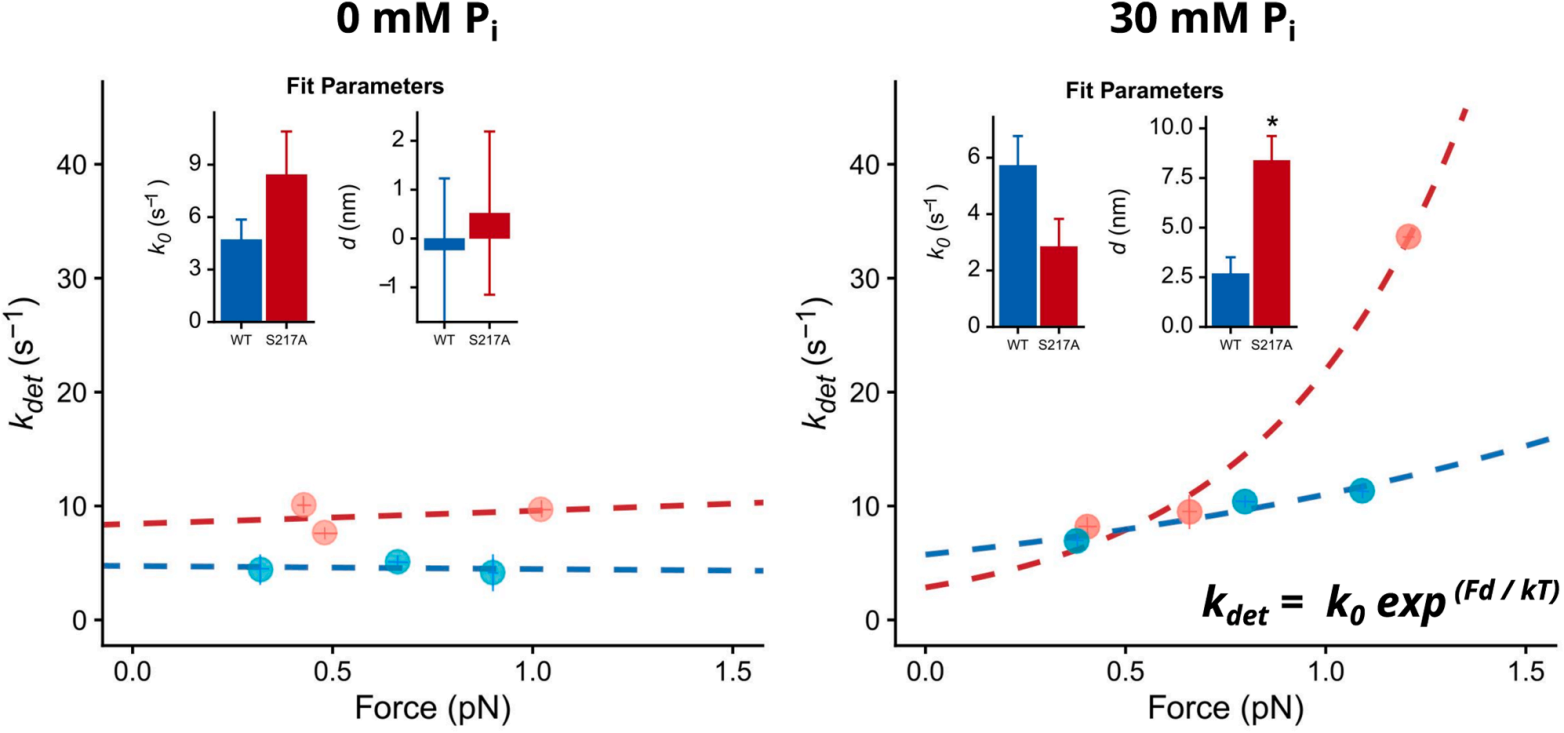
**Load dependence of P_i_-induced detachment for WT and S217A myosin Va.** Applied force vs. the mean ± SEM of the detachment rate (1/*t*_on_) for each trap stiffness, for WT (*blue*) and S217A (*red*) in the absence (*left panel*) and presence of 30 mM P_i_ (*right panel*). Data fit to the Bell equation (shown). Inset, mean ± SEM of parameter estimates. k_0_, rate of detachment in the absence of load and *d*-value the load-sensitive parameter. ∗ indicates significantly different from WT-myosin Va, *p* < 0.05. The *d*-value for WT was also significantly different from zero.

The load-sensitivity of each construct (Fig. 4) was determined by converting the displacements to forces based on the trap behaving as a Hookean spring (*i.e.* Force = *k*_trap_ x displacement) (36, 37). These forces were plotted vs. the detachment rate (1/*t*_on_) and fit to the Bell approximation (38):

$$k_{\det}=k_{0\;}\;\exp^{\left(\frac{Fd}{kT}\right)}$$

where *k*_det_ is the observed detachment rate, *k*_o_ the detachment rate in the absence of force, *F* the force, *k* the Boltzmann constant, *T* the temperature in Kelvin and *d* a fit parameter that quantifies the degree of sensitivity to load in nanometers (33).

At 0 mM P_i_ neither construct’s detachment rate was load-dependent (Fig. 4*A*). However, in the presence of P_i_ the detachment rates of both WT and the S217A were load-dependent, based on the *d* parameter being significantly different from zero. Interestingly, the detachment rates for S217A were three-fold more load-dependent than WT (Fig. 4*B*).

## Discussion

In the present study we sought to determine the order of the powerstroke and P_i_-release by determining if P_i_ release occurs before or after the powerstroke. We took two independent approaches to maintain P_i_ in the nucleotide-binding site while directly measuring the effect on myosin’s powerstroke, hitch and *t*_on_. The first approach was to increase the P_i_ concentration in the experimental buffer to facilitate the rebinding of P_i_ to myosin in the AM.ADP state. The second approach utilized a mutation (S217A) known to slow P_i_ release from the active site (9, 34).

Re-analyzing data from a prior study (8) confirmed that at the lowest trap stiffness 30 mM P_i_ had an insignificant effect on the powerstroke, hitch and *t*_on_ of WT-myosin Va (Fig. 2). This suggests that under these conditions P_i_ rarely rebound to the active site, because P_i_ rebinding is thought to accelerate detachment (6, 24, 39, 40). By contrast, at the intermediate and higher trap stiffnesses, where the cross-bridge experienced higher resistive forces (Fig. 4), *t*_on_ was significantly and progressively reduced by P_i_ (Fig. 2*B*). Therefore, at the highest trap stiffness and 30 mM P_i_, P_i_ likely rebounded so quickly that the AM.ADP.P_i_ state was the predominant biochemical state during the binding event. This idea is further supported by the reduction in *t*_on_ (Figs. 2*B* and 3*B*) because rebinding of P_i_ to AM.ADP putatively accelerates detachment from actin (6, 24, 28, 39).

The ensemble averaging analysis of the WT data indicated that the size and rate of transition to the post powerstroke state were unaffected by 30 mM P_i_, even at the highest trap stiffness (Figs. 2*D* and 3*D*). Computer simulations of binding events (Fig. S2) with an imposed delay before the powerstroke confirmed that if there was even a 2 ms delay before the start of the powerstroke in the experimental data it would have been detected by the ensemble averaging analysis. Specifically, in these simulations, a 2 ms delay prior to the powerstroke decreased the rate of this transition from 7000/s to 200/s. In the experimental data the rate of this transition (k_0_) was >5000/s (Figs. 2*D* and 3*D*) in the presence and absence of P_i_ and for both constructs, despite the significant decrease in *t*_on_ (Figs. 2*B* and 3*B*), which is indicative of P_i_ rebinding to the active site. The decrease in *t*_on_ at the ATP concentration used (100 μM) implies that P_i_ predominantly rebound to actomyosin in an AM.ADP state. This strongly suggests that the powerstroke occurred while P_i_ was bound inside the active site, consistent with a model in which the primary powerstroke precedes the release of P_i_ from the nucleotide-binding site (6, 8, 10, 11, 27).

While the initial powerstroke was unaffected by 30 mM P_i_ at the highest trap stiffness, the secondary powerstroke, or hitch, was effectively eliminated (Fig. 2*D*). This decrease is consistent with a decrease in the percentage of binding events ending before completing the hitch and ADP release, based on the results of simulating binding events (see Supporting Information. This article has Supporting Information. For example, the decrease in the size of the hitch from 1.3 nm to 0.3 nm in the WT construct (Fig. 2*D*) would be consistent with the hitch being prevented in 77% of the binding events observed. Taken together with the reduction in *t*_on_ (Fig. 2*B*), this finding suggests that at 30 mM P_i_ in the majority of binding events myosin detached from the actin filament before undergoing the hitch. The hitch is putatively associated with ADP release from the active site (25, 26); therefore, this suggests that P_i_ release occurs after the powerstroke but prior to ADP release. This order of events is consistent with the order of the biochemical events proposed based on transient kinetic experiments in solution using myosin II (41) and myosin Va (3, 42, 43) as well as other myosins (44). The present study now reveals the order of the mechanical events relative to these biochemical transitions.

The construct containing the S217A mutation provided an independent method of maintaining P_i_ in the active site. This mutation has been shown to slow P_i_-release, up to 10-fold (9, 34, 35) due to disrupted coordination of P_i_ within the active site, and it is specifically thought to slow the exit from the nucleotide-binding pocket (9). Indeed, this mutation was used to support the hypothesis that P_i_ is rapidly released from the active site but stalls in the P_i_-release tunnel before being released into solution (9). Therefore, in the S217A construct P_i_ should remain in the active site for 30-70 ms after binding to actin. The ensemble averaging analysis indicates that myosin transitioned from unbound to strongly bound, post-powerstroke in ∼2 ms (Figs. 2*D* and 3*D*), far faster than the rate of P_i_-release for this mutant construct (9, 34, 35). Therefore, this suggests that the powerstroke occurred while P_i_ remained in the active site. If P_i_-release preceded the powerstroke, both the size and rate of the initial powerstroke should have been reduced, but these measures were unaffected (Figs. 2*D* and 3*D*). Additionally, *t*_on_ would be expected to be prolonged if P_i_-release preceded the powerstroke, rather than being unchanged or reduced as was observed (Fig. 3*B*).

To further extend the time that P_i_ remained in the active site the S217A construct was exposed to 30 mM P_i_. As with WT, P_i_ did not affect the size or rate of the primary powerstroke (Fig. 3*D*). Thus, despite the slowed rate of P_i_ release, the primary powerstroke still preceded P_i_-release in this construct. This implies that the energy that drives the powerstroke is not coming from the release of P_i_, as originally proposed (41), but from the formation of the strong actomyosin bond (45).

Interestingly, the S217A construct displayed a more complex response to increased stiffness than the WT construct (Fig. 3*B* vs, 2*B*). P_i_ did not decrease *t*_on_ in S217A at the low or intermediate stiffnesses but produced a significantly greater decrease at the highest trap stiffness (Fig. 3*B*). Ensemble averaging of these binding events revealed that the hitch was also reduced to near zero (Fig. 3*D*). This suggests that P_i_-release also occurs after the primary powerstroke but before the hitch in the S217A construct. Therefore, despite the alteration in the rates of P_i_-release and ADP-release for this construct, the order of the mechanical and kinetic events remained the same as in WT.

Fitting the detachment rates (1/*t*_on_) vs force data to the Bell-bond equation (38) (Fig. 4) provided quantitative insight into the load-dependence of the P_i_-induced detachment rates (33, 46, 47). In the absence of P_i_ the increased load, presented by higher trap stiffness, did not slow the detachment rate for either construct, in contrast to similar studies with other myosins (33, 46, 47). However, the resistive forces experienced in the present study were lower than those applied in prior investigations (33, 46, 48). This suggests that there may be a minimum threshold required to slow the detachment rate. In contrast, at 30 mM P_i_ the detachment rate was strongly load-dependent in both constructs, getting progressively faster with increasing resistive load (Fig. 4*B*). This is consistent with prior observations suggesting that the rate of P_i_ rebinding is strain-dependent (6, 28, 46). This suggests that the opening of switch II, which is thought to gate P_i_ re-entry into the active site (9, 14, 16, 49), is modulated by resistive load. Interestingly, this analysis also revealed that S217A is significantly more sensitive to load than WT (Fig. 4*B*), implying that the switch I region of the active site, where the mutation lies, is involved the load dependence of P_i_-rebinding.

Although our experiments were done using single-headed myosin Va constructs, the data may have implications for understanding how myosin Va is able to walk processively along an actin filament (3, 4, 43). Specifically, these data suggest that the lead head may transiently exist in a post-powerstroke state with ADP and P_i_ still in the active site (50), because the powerstroke was unaffected by elevated P_i_ (Fig. 2*D*), even in the S217A construct (Fig. 3*D*) where P_i_-release is much slower (9, 34, 35). However, this is likely to be a short-lived state under high resistive strain because elevated levels of P_i_ increased the detachment rate at higher trap stiffnesses (Figs. 2*B* and 3B). The lead head would likely be the first to detach because it is under higher strain (50, 51). This implies that the predominant state near stall force is a strained AM.ADP state, which is consistent with how dimeric myosin Va processes against the force of a laser trap in the presence of elevated levels of P_i_ (28).

In conclusion, these findings are most consistent with a model of myosin’s ATPase cycle in which the primary powerstroke precedes P_i_-release from the active site (8, 10, 11, 27, 52). However, it also suggests that P_i_-release precedes the smaller secondary powerstroke or hitch. Thus, these data suggest an order for the biochemical and mechanical transitions by myosin Va that are key to understanding how this prototypical molecular motor transduces the chemical energy from the hydrolysis of ATP into the mechanical work in the form of the powerstroke and hitch.

## Experimental procedures

### Proteins

Single-headed WT and S217A mutant myosin Va S1 constructs were expressed and purified using methods previously detailed (34). The expressed proteins were purified with FLAG affinity chromatography. Actin filaments were polymerized from actin isolated from chicken pectoralis muscle as previously detailed (53, 54). After polymerization the actin filaments were labeled with an equimolar ratio of TRITC/phalloidin and biotin/phalloidin.

### Single-molecule laser trap assay

A three-bead single molecule laser trap assay (Fig. 1) was used to determine the size of the powerstroke and the duration of the strongly-bound lifetime of each actomyosin bond (*t*_on_) as previously detailed (55). The control experimental buffer included an oxygen scavenging system (29 mM of glucose, 1.5 mM glucose oxidase, and 80 units catalase), as well as 91 mM KCl, 1 mM EGTA, 4 mM MgCl_2_ and 1 mM DTT at pH 7.0, 25^o^C and 100 μm ATP. For experiments in the presence of 30 mM added P_i_, the concentration of KCl was reduced to maintain the total ionic strength of 125 mM.

The three-bead assay was performed as previously described (6, 19), as shown in Figure 1. The x-axis position of one of the trapped beads was recorded from the quadrant photodiode at sampling rate of 5 kHz. The system was calibrated by moving a fiduciary silica bead on the coverslip a known distance, as previously described (55). The stiffness of bead–actin–bead assembly was determined using the equipartition method (56). The control data, obtained at a trap stiffness of 0.04 pN nm^−1^, were taken from a previously published study (8), with permission from the authors. In the present study, we compared these data with experiments performed at trap stiffnesses of 0.06 and 0.10 pN nm ^−1^.

### Data analysis

Displacement data were analyzed with custom programs written in *R* as previously described (8), using a two-state Hidden-Markov Model (HMM) algorithm (57, 58) to identify actomyosin binding events and changepoint analysis (59) to determine precise estimates of the start and end of each events. *t*_on_ was defined as the time between the start and end of each event as identified by the changepoint analysis. The magnitude of the displacement or powerstroke was determined as the difference between the average displacement during a binding event and the average position immediately before the start of the binding event.

Ensemble averaging of the actomyosin binding events was done by aligning the start and end of the binding events and then extending the number of points to equalize event durations (Figs. 2*D* and 3*D*) (8). Forward ensembles were then fit with a double exponential equation (y = *d*_*1*_*∗(1-exp(x∗-k*_*0*_*)) + d*_*2*_*∗(1-exp(x∗-k*_*1*_*)))* and the backward ensembles were fit to a single exponential equation (*y = d*_*1*_ *+ d*_*2*_*∗exp(x∗k*_*2*_*)*), where *d*_*1*_ represents the magnitude of the displacement for the primary powerstroke, *k*_*0*_ and *k*_*1*_ rates of transition into the powerstroke, *d*_*2*_ the size of the hitch and *k*_*2*_ the rate of detachment from the binding event (8, 33, 60).

### Inferential statistics

Distributions of *t*_on_ were non-normally distributed (Kolmogorov-Smirnov, *p* < 0.05); therefore, the data were analyzed using non-parametric methods. An *Aligned Rank Transformation* (61), (https://cran.r-project.org/web/packages/ARTool/index.html) was used to enable running a three-way ANOVA (Myosin construct x P_i_ concentration x trap stiffness) followed by a Tukey’s *post hoc* test with a Bonferroni adjustment for multiple comparisons, with an α-level of 0.05. Differences in the Bell equation parameters were determined by constructing 95% confidence intervals around the parameter estimates.

## Data availability

The raw and analyzed data will be made available upon request to the corresponding author, E. P. D.

## Supporting information

This article contains supporting information (8, 33, 57).

## Conflict of interest

The authors declare that they have no conflicts of interest with the contents of this article.
