# Supplementary material for: Phosphate release from myosin Va occurs after the initial powerstroke but before the secondary powerstroke associated with ADP-release[image]
Source: J Biol Chem. 2026 Jun 29;302(8):113303. doi: 10.1016/j.jbc.2026.113303 (PMC13452157; doi:10.1016/j.jbc.2026.113303)
Supplement: Supplementary file 1 [file mmc1.docx]

**Supplemental Materials**

**A. Results of simulations of decreasing the probability of the hitch**

We interpreted the decrease in the size of the hitch in the presence of P_i_ as a decrease in number events that completed the secondary powerstroke, or “hitch”, which is associated with ADP-release. In order to test our interpretation that the decrease in the size of the hitch, as measured in the ensemble averaging analysis, can be explained by a decrease in the number of single molecule binding events that progressed through a second powerstroke, we generated a series of simulations replicating single molecule optical trapping data (Fig. S1 A). We first simulated a control condition where 500 binding events were simulated in which the primary powerstroke (d1) was set to 6 nm and the hitch (d2) to 1 nm (Fig. S1 B). In the first set of simulations, 100% of the events completed a hitch, serving as the control condition. We then generated additional data sets in which the number of events that completed a hitch was reduced to 75%, 50%, 25% and finally 0% (Fig. S1 A). A total of 500 binding events were generated for each condition, therefore the number of events that completed a secondary powerstroke were progressively decreased from 500, to 375, to 250, to 125 and finally to 0.

These simulations were performed using an algorithm within our custom analysis software package, *lasertrapr,* which is distributed as shareware within the R-package (see Methods). The baseline position was set at 0 nm, with a superimposed Gaussian noise profile with a standard deviation of 8 nm. The addition of the Gaussian noise reproduced the “Brownian capture” of binding events observed in the experimental data. During a bound event there was also a Gaussian noise profile that was added around the displacement which was specified to have a 4-fold reduction in noise to replicate the reduction in noise when myosin binds to the bead-actin-bead assembly. These noise, and displacement, characteristics were chosen based on the noise characteristics of the experimental data observed using our laser trap instrumentation (see e.g. displacement records in Figure 2, main text). These specific characteristics replicate the Brownian motion of an optically trapped 1 micron silica microsphere at a laser trap stiffness of 0.04 pN^.^nm^-1^.

The resultant simulated data were analyzed with the same analysis program and method as the experimental data, using a combination Hidden-Markov model(3) and changepoint analysis(4). After event identification, the start and end of each detected binding event were temporally aligned for ensemble averaging. The forward ensemble averages were fit to the following equation:

$$y=d1*\left( 1-exp\left[ -ko*x \right) \right]+d2*(1-exp\left[ -k1*x \right]))$$

where *d1* is the primary powerstroke, *d2* the hitch associated with ADP-release, *k_0_* the rate of transition from the unbound to post-powerstroke state, *k_1_* the rate of transition through the hitch.

The backwards ensemble averages were fit to:

$$y=d1+d2*exp\left[ x*k2 \right])$$

where *d_1_* and *d_2_* are the primary powerstroke and hitch respectively and *k_2_* is the rate of exiting the bound state (i.e. the detachment rate).

Figure S1 A shows that as the number of binding events that complete the hitch is reduced the magnitude of the hitch observed in the ensemble averages is progressively reduced. Moreover, the magnitude of the hitch is directly correlated with the percentage of events that generate a hitch (Fig. S1 C). These simulations therefore are consistent with the decrease in the size of the hitch resulting from a decrease in the percentage of single actomyosin interactions that complete a hitch

Figure S1.

**B. Simulations of a delayed rate of transition from a weakly/unbound state to the strongly-bound post powerstroke state**

Computer simulations of actomyosin binding events were performed to determine if the ensemble averaging analysis could detect a delay in the rate of the powerstroke. The same software, algorithm, and noise characteristics used were identical to those described in Supplementary section A (above). For this set of simulations 500 binding events were generated having a primary powerstroke of 6nm and a hitch or substep of 1nm. Three distinct simulations were run 1) the powerstroke occurring immediately upon strongly-binding to actin, 2) the powerstroke occurring after a 2 ms delay, and 3) the powerstroke occurring after a 5 ms delay. The events were temporally aligned and subjected to the ensemble averaging analysis. For the events without a delay the rate of transition (*k*_o_) was >5000/s while the 2 ms delay decreased this rate to 241/s, and it was further reduced to 196/s with a 5 ms delay (Fig. S2 A). Examination at a shorter time scale demonstrates the difference in rates (Fig. S2 B). The table in Figure S2 (S2 C) demonstrates that this delay would also be apparent as an increase in the size of the secondary powerstroke or substep. These data therefore suggest that if P_i_ or the presence of the S217A mutation delayed myosin Va’s powerstroke the ensemble average analysis would have been able to detect this change. Since *k*_o_ exceeded 5000/s for all conditions and constructs in the experimental data (Figs. 2 and 3 main text) this suggests that P_i_ and the S217A mutation did not prevent or delay the powerstroke.

Figure S2

**References**

1.	Veigel, C., Molloy, J. E., Schmitz, S., and Kendrick-Jones, J. (2003) Load-dependent kinetics of force production by smooth muscle myosin measured with optical tweezers. *Nat Cell Biol*. **5**, 980–986

2. https://github.com/brentscott93/lasertrapr

3.	Smith, D. A., Steffen, W., Simmons, R. M., and Sleep, J. (2001) Hidden-Markov Methods for the Analysis of Single-Molecule Actomyosin Displacement Data: The Variance-Hidden-Markov Method. *Biophysical Journal*. **81**, 2795–2816

4.	Scott, B., Marang, C., Woodward, M., and Debold, E. P. (2021) Myosin’s powerstroke occurs prior to the release of phosphate from the active site. *Cytoskeleton (Hoboken)*. **78**, 185–198
